# Preparation and Performances of Polyether Polytriazole Elastomers Based on Click Chemistry

**DOI:** 10.3390/polym14173538

**Published:** 2022-08-29

**Authors:** Kun Cong, Zhenhui Liu, Fa Hu, Jiyu He, Rongjie Yang

**Affiliations:** 1 China Petroleum Engineering & Construction Corporation Beijing Company, Beijing 100085, China; 2 School of Materials Science and Engineering, Beijing Institute of Technology, Beijing 100081, China; 3 SINOPEC (Beijing) Research Institute of Chemical Industry Co., Ltd., Beijing 100013, China

**Keywords:** PTPET, twin-screw reactor, flame retardant, composite

## Abstract

Since the polyurethane elastomer synthesis process is susceptible to moisture, polytriazole polyethylene oxide-tetrahydrofuran (PTPET) elastomer was used as a replacement owing to its mild production environment. In contrast to the conventional flask-synthesis method, the twin-screw reactor instrument could provide more meaningful data in the synthesis. In this study, PTPET elastomer was prepared by the MiniLab twin-screw reactor method for the first time, and the activation energy of the PTPET elastomer was calculated using the torque variation obtained from the MiniLab twin-screw reactor during the synthesis process at two different temperatures. The addition of flame retardants could endow the composites with more useful properties. The PTPET composites poly (phenylsilsesquioxane) (PTPET-PPSQ), octaphenyl polyhedral oligomeric silsesquioxane (PTPET-OPS) and PTPET-PhVPOSS (phenyl/vinyl polysilsesquioxane) were synthesized by using the MiniLab twin-screw reactor. The prepared PTPET elastomer and composites were fully characterized by FT-IR, TG, DSC, swelling test, mechanical test, SEM and combustion test. The characterization results show that the addition of the flame retardants has little influence on the original structure and properties of PTPET elastomer. The flame retardancy was characterized by the combustion test showing that all PTPET composites form a certain thickness of char layer during the burning process. These results indicate that the addition of flame retardants maintains the outstanding properties of PTPET elastomer and also endows the materials with a certain extent of flame retardancy; thus, it is believed to be a good engineering material that could be applied in many realms.

## 1. Introduction

The adhesive systems of the binder ethylene oxide-tetrahydrofuran copolymer (PET) [1] and hydroxyl-terminated polybutadiene (HTPB) [2,3,4,5] with isocyanate as curing agent (methylene diphenyl diisocyanate (MDI), toluene diisocyanate (TDI), etc.) are polyurethane adhesive systems commonly used in composite solid propellants. Polyurethane adhesive systems are sensitive to moisture, and the presence of moisture seriously affects the cross-linking network structure and reduces the mechanical strength of the polyurethane elastomers [6,7,8]. Currently, based on the click chemistry method [9,10,11], the new curing system of alkynyl-terminated polyether and multifunctional azide compounds is not affected by moisture in the air compared with the polyurethane curing system, and the main chain of alkynyl-terminated polyether also maintains good flexibility as its repeat unit is the same as hydroxyl-terminated polyether, showing excellent low-temperature performance and other advantages. Therefore, it is believed to be a better new solid propellant binder.

Click chemistry is an efficient method for the rapid synthesis of various compounds by linking characteristic chemical groups together through a mild and high-yield reaction. Its purpose is to obtain diverse molecules by means of click chemistry based on the synthesis of carbon heteroatoms C-X-C [12,13]. A representative reaction of click chemistry is the copper-catalyzed azide-alkynyl Huisgen cycloaddition [14,15,16,17]. Through a 1,3-dipolar cycloaddition reaction, the azide group and the alkynyl group are transformed into a compound of a 1,2,3-triazole structure [18,19]. The reaction process is simple and rarely affected by the environment. The polyether polytriazole prepared by alkynyl–azide click reaction has attracted attention in the development of composite propellants [20,21,22]. Meanwhile, flame retardants could be added to extend the applications of the polytriazole materials, and various methods and instruments could be used to achieve this objective.

In this study, the MiniLab twin-screw reactor [23] was used to prepare polytriazole polyethylene oxide-tetrahydrofuran (PTPET) elastomer from alkynyl-terminated polyethylene oxide-tetrahydrofuran (ATPET) and glycidyl azide polymer (GAP). The oligomeric silsesquioxane compounds PPSQ, OPS and PhVPOSS [24,25] were used as functional additives to prepare PTPET elastomer composites PTPET-PPSQ, PTPET-OPS and PTPET-PhVPOSS. The prepared PTPET-PPSQ, PTPET-OPS and PTPET-PhVPOSS were fully characterized. At the same time, based on the torque parameters obtained in the reaction process from the MiniLab twin-screw reactor, the kinetic model data of the PTPET elastomer were calculated.

## 2. Experimental Section

### 2.1. Materials

**Figure 1:**
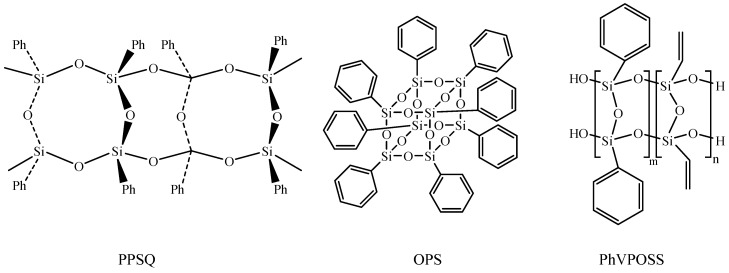
PPSQ, OPS and PhVPOSS, silsesquioxanes with different structures.

**Figure 2:**
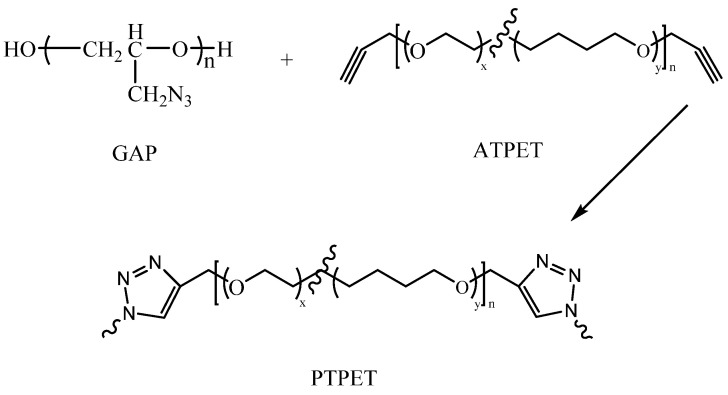
The click chemistry reaction between GAP and ATPET to synthesize PTPET.

ATPET (Mn = 4000 g/mol, alkyne average functionality of 2) was obtained from the Beijing Institute of Technology (Beijing, China). GAP (Mn = 480 g/mol, -N_3_ average functionality of 3.82) was purchased from Liming Research & Design Institute of Chemistry (Luoyang, China). Tetrahydrofuran was purchased from Beijing Tongguang Fine Chemicals Company (Beijing, China). PPSQ, OPS and PhVPOSS were synthesized by Beijing Institute of Technology (Beijing, China). Their structures are depicted in Figure 1. A kind of organic copper(I) complex C2610 was purchased from J&K Scientific Ltd (Beijing, China). The click chemistry reaction is shown in Figure 2.

### 2.2. Preparation of PTPET Elastomer and Composites

Two raw materials, ATPET and GAP were dried in two separate beakers in a vacuum oven at 80 °C for 2 h to remove moisture before use. Then, 0.25 wt % organic copper(I) complex C2610 was dissolved in 1 mL THF and kept in another beaker. The polymerization process was carried out in a small-scale conical twin-screw extruder (Haake MiniLab) with two screws (length of 11 cm) and a capacity of around 10 g. The temperature of the cavity of the extruder was set as 80 °C and 90 °C, respectively, to prepare PTPET and study the kinetic behavior of the polymerization. Screw rotation speed and direction are key factors for the reaction since they provide the necessary shear force for the reaction. The screw rotation speed was set at 90 rpm and the rotary direction was set opposite to each other. The twin-screw extruder could control the fed materials either being cycled in the cavity or extruded during the whole reaction process through a key semilunar switch. The reaction started after the raw materials had been mixed and fed into the extruder. The reaction was monitored by the torque value given by the apparatus.

PPSQ, OPS and PhVPOSS were dried in the oven at 80 °C in vacuum for the removal of moisture. Three sets of ATPET (Mn = 4000 g/mol, 15 g, 3.75 mmol) and GAP (Mn = 480 g/mol, 0.76 g, 1.58 mmol) mixtures were prepared in three separate beakers before use. Then, 1.6 g (10 wt %) of PPSQ, OPS and PhVPOSS was added to these three beakers, respectively. Three sets of 0.25 wt % organic copper(I) complex C2610 were dissolved in 1 mL THF and added to these three separate beakers, respectively. The screw rotation speed was set at 90 rpm and the temperature in the cavity of the extruder was set to 80 °C. The rotation direction of the two screws was set as counter-rotating.

Detailed feeding amounts of each raw material are listed in Table 1.

### 2.3. Measurements and Analysis

#### 2.3.1. FT-IR Analysis

FT-IR spectra were used to verify the raw materials and the products. Measurements were performed on a Nicolet AVATAR 6700 FT-IR spectrometer (ThermoFisher SCIENTIFIC, Waltham, MA, USA). FT-IR spectra were scanned with a resolution of 4 cm^−1^ in the range of 400 cm^−1^ to 4000 cm^−1^ 32 times per measurement.

#### 2.3.2. Mechanical Properties Analysis

The mechanical properties of PTPET and PTPET composites were measured by a tensile strength apparatus (MTS SYSTEM, Beijing, China). All the mechanical data including tensile strength and strain were the average of five tests. The test samples of PTPET and PTPET composites were cut from a Teflon module 8 cm long, 5 cm wide and 4 mm thick into suitable size based on GB/T 528 for the mechanical tests.

#### 2.3.3. Differential Scanning Calorimetry (DSC) Analysis

DSC tests were performed on a Netzsch Instruments DSC 204 F1 Phoenix (Netzsch, Germany). The temperature range for operating the measurements was between −100 °C and 220 °C. The detailed procedure for performing the DSC experiments was as follows: Usually, around 5 mg of the PTPET and PTPET composites samples were loaded into the bottom of the aluminum crucible and pressed hard with a cover before being put into the DSC apparatus. The PTPET and PTPET composites were heated from room temperature to 220 °C with a rate of 10 °C/min to remove the previous thermal history. They were then cooled to −100 °C with liquid nitrogen at a rate of 20 °C and then heated back to 220 °C at a rate of 10 °C/min.

#### 2.3.4. Thermogravimetry (TG) Analysis

Thermogravimetry analysis was performed on a Netzsch 209 F1 (Netzsch, Germany, Al_2_ O_3_ crucible) instrument. Samples were tested in the atmosphere of N_2_. The scanning rate was 10 °C/min and the temperature range was between 40 °C and 700 °C.

#### 2.3.5. Fire Testing Method

Cone calorimeter data were obtained using a Fire Testing Technology device based on ISO 5660 protocol (FTT, Derby, UK). Samples prepared with the dimensions of 100 mm × 100 mm × 3 mm were measured horizontally without any grids and exposed to a radiant cone of 50 kW/m^2^. Typically, the results were reproducible with an undulation of ±10%.

## 3. Results and Discussion

### 3.1. PTPET Elastomer

PTPET elastomers could be prepared via a click chemistry reaction using ATPET and GAP as the raw materials. GAP is a small molecule with an average of 3.8 -N_3_ groups, and ATPET has two terminal alkyne groups. When the azido and alkynyl groups are equimolar, a cross-linking structure will be formed. Molecular weights and cross-linking extents of the elastomers are controlled to keep the torque of the polymerization system bearable by the twin-screw extruder.

**Figure 3:**
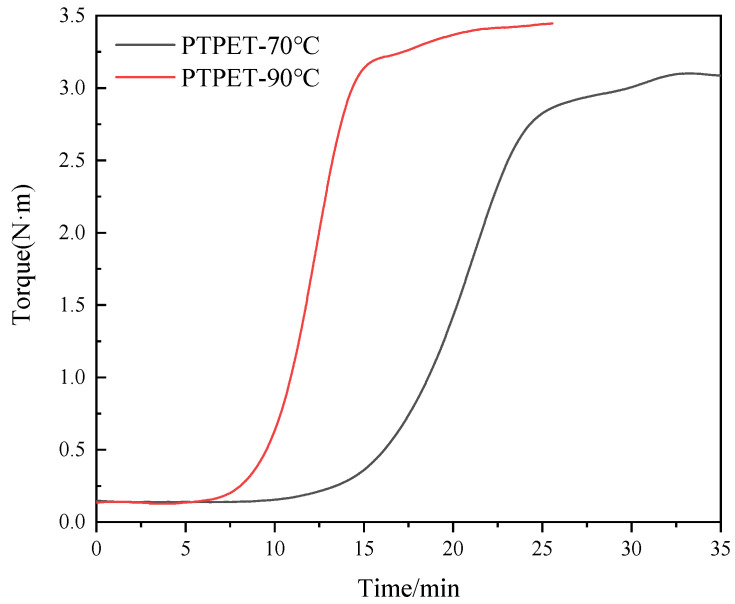
Torque versus time of the reaction between ATPET and GAP inside the twin-screw extruder.

The torque is a significant parameter in the reaction process that is largely affected by the reaction temperature, the molecular weights of the products and the cross-linking extents. The reaction extent of the click chemistry inside the twin-screw extruder can there be estimated by studying the changes in torque during the reaction process. Figure 3 shows the variation of the torque with time inside the MiniLab twin-screw reactor during the whole click chemistry reaction process between ATPET and GAP. This figure exhibits S-shaped curves with three clear regions: The first stage is the heating stage of the reactant mixture ATPET, GAP and the catalyst. For the reaction performed at 70 °C, the reaction started after 10 min. When the reaction was set to 90 °C, it could be observed that the torque of the reactant mixture started to increase in about 6 min. In the second stage, the torque started to increase significantly since in this period the polymerization reaction reached its maximum rate. At 70 °C, it could be observed that the rapid reaction period lasted for about 15 min and the reaction rate began to decline after 25 min. At 90 °C, the reaction process exhibits similar characteristics, while the rapid reaction period lasted for a shorter time, only about 5 min. In the third stage, the torque of the reaction material was at a high level, and the polymerization reaction tended to be completed. As the contents of the reactive groups decreased, the reaction rate declined as well.

It could also be observed from the torque–time curve in Figure 3 that in the late period of the polymerization reaction, the torque of the reaction system has a small upward step. This phenomenon may be related to the transformation of PTPET from a linear structure to a cross-linking structure, which needs further study.

The torque curve in Figure 3 could be used in the analysis of chemical reaction kinetics. The percentage of conversion, *α*, could be converted from torque, *T(t)*, given as the following Equation (1):

(1) $$\alpha =\frac{T(t)-T_{t=0}}{T_{t\to \infty }-T_{t=0}}$$

In this equation, $T_{t=0}$ is the starting torque when raw materials are just added into the twin-screw extruder, while $T_{t\to \infty }$ represents the value of torque when the click chemistry reaction finishes. Usually, at this step, the torque could be recognized as a stable plateau in the curve. The slightly excess amount of bifunctional ATPET ensures that the reaction between the alkyne and azide approaches the extent of 100%.

The conversion rate of the reaction could be expressed as the following Equation (2):

(2) $$\frac{d\alpha }{dt}=k\cdot f(\alpha )$$

where *k* is the reaction kinetic rate constant and *f*(*α*) refers to a function with the conversion as the independent variable *α*. The kinetic rate constant *k* follows the Arrhenius Equation (3):

(3) $$k=A\cdot e^{\frac{-E}{RT}}$$

where *A* is the frequency factor, *E* is the activation energy, *R* is the universal gas constant and *T* is the thermodynamic temperature. The combination of the equations is expressed as the following Equation (4):

(4) $$\frac{d\alpha }{dt}=A\cdot e^{\frac{-E}{RT}}\cdot f(\alpha )$$

The relative parameter *E* could be solved by conducting the reaction at two distinct temperatures. In this study, the click chemistry reaction was conducted at 70 °C and 90 °C, which are suitable for a mild reaction process. The curves of the conversion rate *α* versus time *t* are fit by a growth/sigmoidal type Solgistic 3 function via the Levenberg–Marquardt method. The simulated reaction model is given as the following equation:

(5) $$y=\frac{a_{1}}{1+b_{1}e^{-k_{2}x}}$$

**Figure 4:**
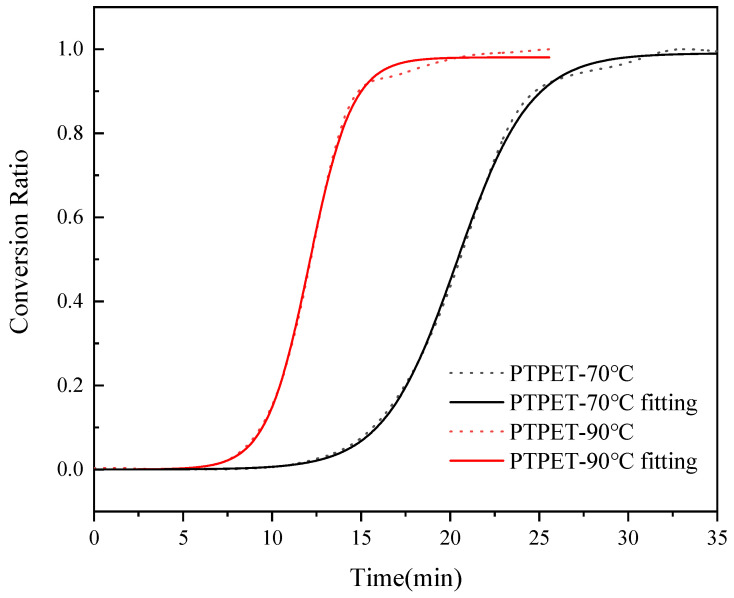
The conversion ratio of ATPET and GAP reactions versus time and the fitting curve.

At 70 °C, *a*_1_ = 0.99018, *b*_1_ = 19,635.119 and *k*_2_ = 0.48509; when the reaction proceeds at 90 °C, *a*_1_ = 0.98056, *b*_1_ = 21555.506 and *k*_2_ = 0.8255. The curve and fitting of conversion rate *α* versus time *t* are shown in Figure 4. The kinetic fitting equation perfectly matches the curve of conversion rate *α* versus time *t*, which can reflect the click chemistry reaction process of preparing PTPET elastomer in a MiniLab twin-screw reactor. The activation energy could be calculated as 27.01 kJ/mol. The click chemistry reaction could hardly occur without catalyst at 70 °C or 90 °C. Therefore, it is believed that the addition of organic copper(I) complex C2610 decreases the activation energy of the click chemistry reaction.

### 3.2. PTPET Composites

**Figure 5:**
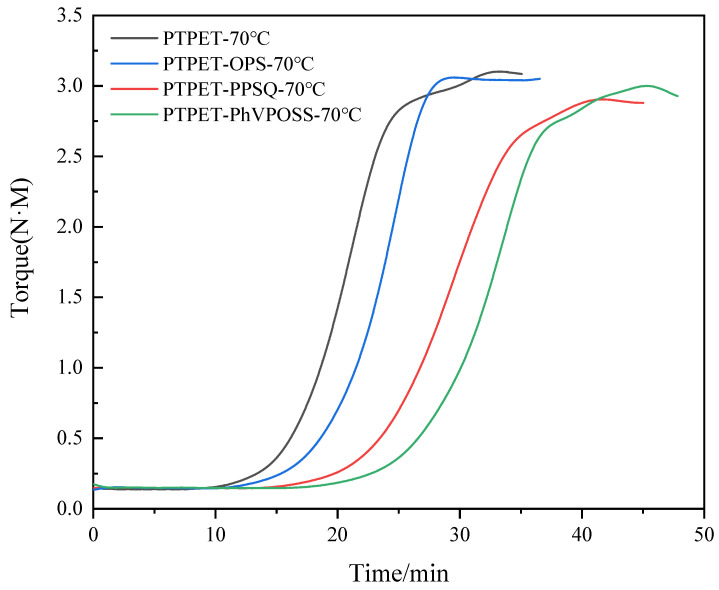
Torque versus time curves of PTPET elastomer and composites at 70 °C.

**Figure 6:**
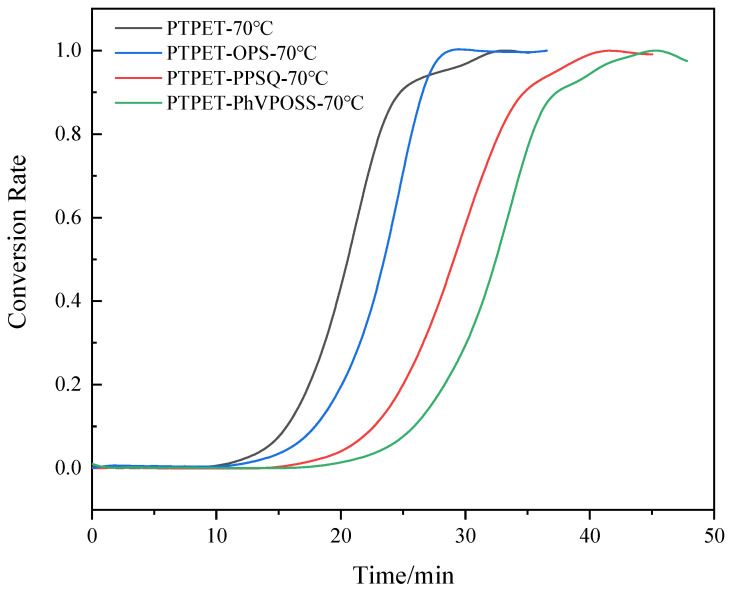
Conversion rate versus time curves of PTPET elastomer and composites at 70 °C.

In order to study the influence of different additives on the click chemistry reaction between azide and alkyne, torque versus time curves for PTPET, PTPET-PPSQ, PTPET-OPS and PTPET-PhVPOSS prepared at 70 °C are shown in Figure 5. Figure 6 shows the conversion rate–time curve of PTPET elastomer and three composites. Compared to the process of preparing PTPET elastomer, all three click chemistry reactions with additives generally exhibited similar S-shaped curves during the entire reaction process. The click chemistry reactions could be clearly seen to have three stages: In the initial stable stage, the raw materials ATPET, GAP, catalyst C2610 and flame retardant are heated and sheared for a better mixing condition. Differences in the rate of temperature increase could be clearly observed after the additives were added. The system with OPS has the highest rate of temperature increase. The middle reaction accelerating stage where the torque increases significantly indicates the most rapid click chemistry reaction between ATPET and GAP. It could be observed that the click chemistry reaction is barely affected by the additives since all three systems could still react rapidly. The final stable stage where the torque values remain at a high level indicates that the reaction approaches the end. The torque as the parameter of the reaction extent could be used to compare the reaction with different additives. For PTPET-OPS, the accelerating period is dramatically steep, and the reaction is completed in 27 min and remains stable as the click chemistry approaches the end. The polymerization of PTPET-PPSQ and PTPET-PhVPOSS composites was delayed compared to that of the PTPET-OPS. At the end of the polymerization, PTPET-PPSQ and PTPET-PhVPOSS composites seem to degrade according to the reduction in torque.

### 3.3. FT-IR Analysis

**Figure 7:**
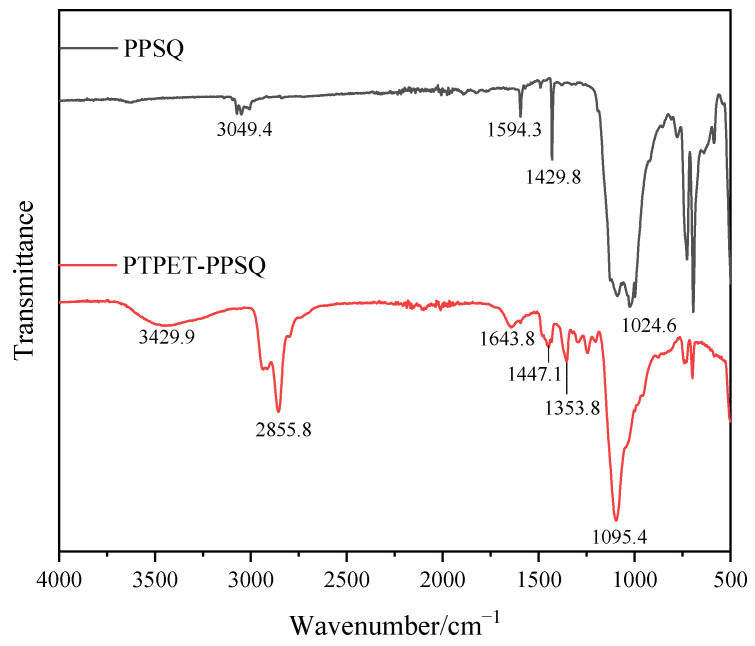
FT-IR analysis of PTPET-PPSQ.

The FT-IR spectrum of the prepared PTPET-PPSQ is shown in Figure 7. It can be seen from the FT-IR of PPSQ in Figure 7 that there is an unobvious absorption band of the Si-OH group that is located within 3200~3700 cm^−^^1^. The absorption peak at 3049.4 cm^−1^ is the stretching vibration peak of C-H in the phenyl group from the PPSQ particles. The absorption peaks at 1594.3 cm^−1^ and 1429.8 cm^−1^ are carbon skeleton vibrations in the phenyl group. The two absorption peaks at 693.5 cm^−1^ and 726.0 cm^−1^ in the fingerprint region are C-H out-of-plane bending vibration regions. The absorption peak at 1095.4 cm^−1^ is the stretching vibration peak of Si-O-Si in PPSQ.

It can be seen from the FT-IR of PTPET-PPSQ that due to the low contents of PPSQ in PTPET-PPSQ, the main characteristic peaks of PPSQ overlap with those of PTPET, so the characteristic peaks of PTPET are mainly displayed. The absorption peak at 3429.9 cm^−1^ is the absorption peak of the -OH from the GAP segments, while the absorption peaks at 2937.0 cm^−1^ and 2855.8 cm^−1^ are the antisymmetric and symmetric stretching vibration peaks of -CH_2_ in PTPET-PPSQ. The absorption peaks located at 1643 cm^−1^, 1447 cm^−1^ and 740 cm^−1^ are the absorption peaks of triazole formed by click chemistry. The azide groups could not be observed in PTPET-PPSQ.

**Figure 8:**
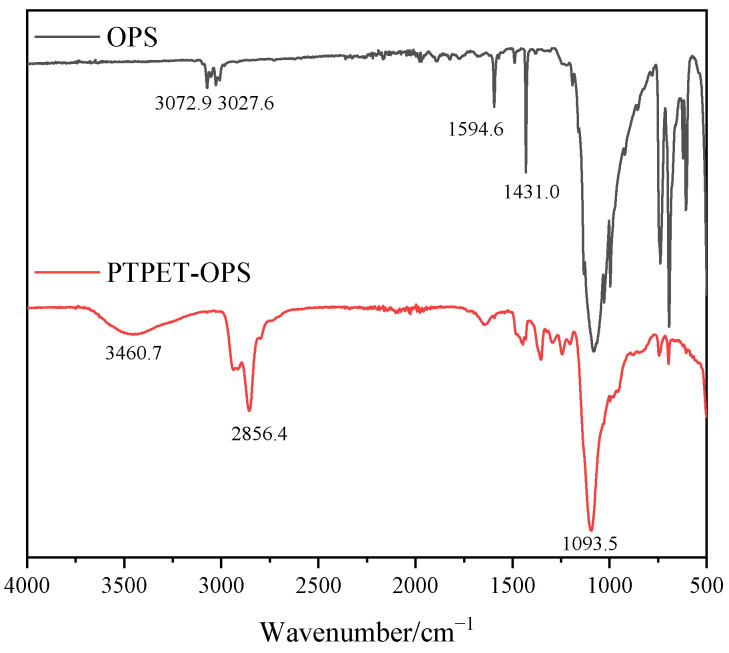
FT-IR analysis of PTPET-OPS.

The FT-IR spectrum of the prepared PTPET-OPS is shown in Figure 8. The absorption peaks at 3072.9 cm^−1^ and 3027.6 cm^−1^ are the stretching vibrations of C-H from the phenyl group in OPS. The absorption peaks at 1594.6 cm^−1^ and 1431.0 cm^−1^ are the absorption peaks generated by the stretching vibration of the carbon skeleton in the benzene ring. The absorption peaks located at 697.1 cm^−1^ and 738.4 cm^−1^ are the out-of-plane bending vibration peaks of C-H in the phenyl group affected by substituents. The absorption peak at 1093.5 cm^−1^ is the stretching vibration peak of Si-O-Si in OPS. It could be seen in the FT-IR of PTPET-OPS composites that most of the characteristic peaks belonging to OPS overlap with those of PTPET. Meanwhile, due to the low amounts of OPS added, the FT-IR of PTPET-OPS composites mainly shows the characteristic peaks of PTPET. For example, 3460.7 cm^−1^ is the absorption peak of -OH mainly from glycidyl azide polymer (GAP), which is the raw material used to prepare PTPET. Since the preparation of the PTPET elastomer and composites involves the click chemistry reaction rather than the reaction consuming the -OH group, the -OH group is therefore retained in the structure. The azide groups could not be observed in PTPET-OPS.

**Figure 9:**
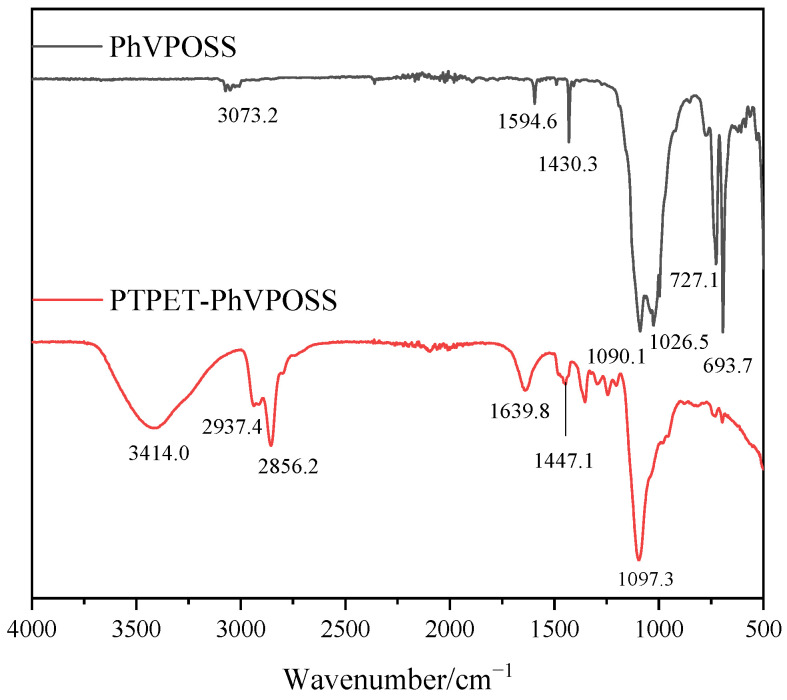
FT-IR analysis of PTPET-PhVPOSS.

The FT-IR spectrum of the prepared PTPET-PhVPOSS is shown in Figure 9. The absorption peak at 3073.2 cm^−1^ is the stretching vibration peak of the =C-H group in the benzene ring. The absorption peaks at 1594.6 cm^−1^ and 1430.3 cm^−1^ are the stretching vibration peaks of the C=C carbon skeleton in the benzene ring. The absorption peaks at 1026.5 cm^−1^ and 1090.1 cm^−1^ are the absorption peaks of Si-O-Si. Due to the induced effect of phenyl and vinyl groups in the surrounding chemical environment, Si-O-Si appears as double peaks in FT-IR. The absorption bands at 693.7 cm^−1^ and 727.1 cm^−1^ are the out-of-plane bending vibration peaks of the vinyl group and C-H in the benzene ring. For PTPET-PhVPOSS composites, except for the -OH group at 3414.0 cm^−1^ and the antisymmetric and symmetric stretching vibration peaks of -CH_2_ at 2937.4 cm^−1^ and 2856.2 cm^−1^ that exist in the PTPET structure, there are still stretching vibration peaks that represent the structure of Si-O-Si from PhVPOSS.

### 3.4. Thermal Analysis

#### 3.4.1. TG Analysis

**Figure 10:**
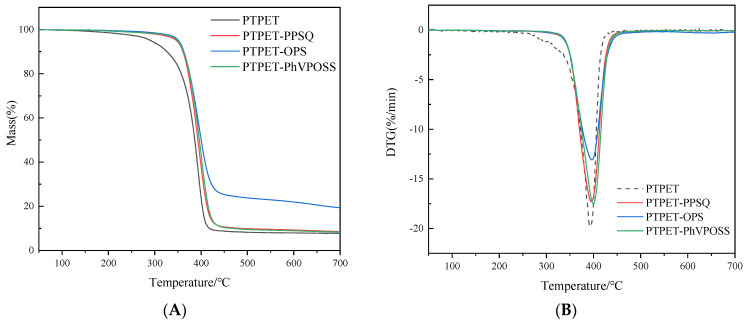
TG (**A**) and DTG (**B**) curves of PTPET elastomer and composites.

The thermal properties of the PTPET elastomer and composites (PTPET-PPSQ, PTPET-OPS and PTPET-PhVPOSS) are key parameters in judging whether the materials are suitable for engineering applications. TG and DTG data of the PTPET elastomer and composites are drawn in Figure 10. Detailed data are listed in Table 2. It could be observed that under the nitrogen atmosphere, the PTPET-OPS composite has the highest thermal stability. The decomposition temperature of the initial 5% (*T*_5%_) total mass is 351 °C. The maximum thermal mass loss rate is at 396 °C, and it has the most residual mass, reaching 19.33% [26]. For PTPET-PPSQ and PTPET-PhVPOSS, both have less residual mass percentage than half of that of PTPET-OPS. The *T*_5%_ decomposition temperature of PTPET elastomer is only 292 °C. The thermal stabilities of the PTPET composites prepared by adding PPSQ, OPS and PhVPOSS have been significantly improved. The maximum thermal decomposition rate temperature of the three PTPET composites is close to 400 °C.

#### 3.4.2. DSC Analysis

**Figure 11:**
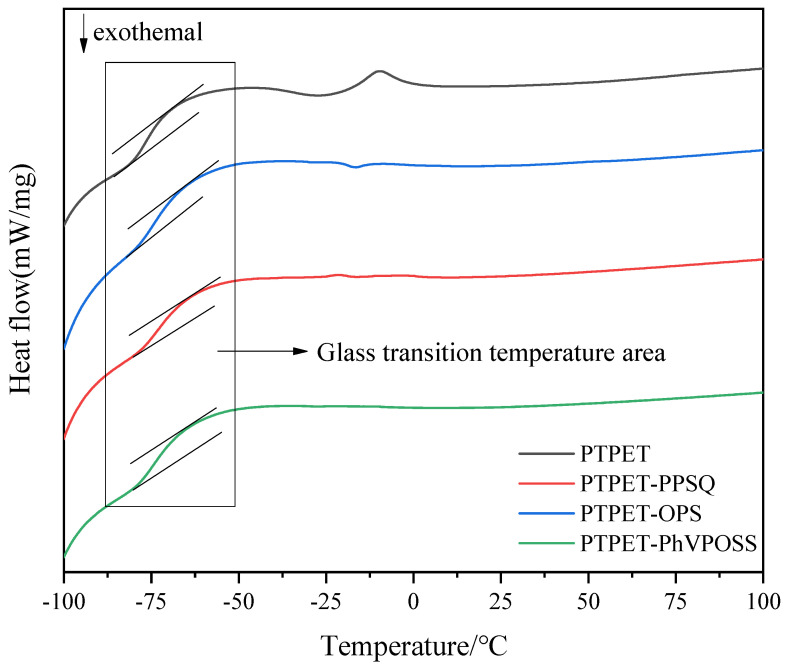
DSC curves of PTPET elastomer and composites.

The heat flux curves from DSC measurements of the PTPET elastomer and composites are shown in Figure 11. Detailed DSC data are listed in Table 3. The box area in Figure 11 is the glass transition region of PTPET elastomer and composites. The glass transition temperature has been identified as the inflection point between two parallel tangents drawn on the curves. It can be seen that there is no significant difference in the glass transition temperature (*T_g_*) between the PTPET elastomer and composites. The *T_g_* of the PTPET elastomer is −76 °C, and the *T_g_* values of all PTPET composites are slightly higher than the *T_g_* of the PTPET elastomer. Among all three PTPET composites, PTPET-PPSQ has a higher *T_g_*, namely −72 °C. The *T_g_* of PTPET-PhVPOSS is lower, namely −75 °C. When comparing all the *T_g_* values shown in Table 3, it can be seen that the *T_g_* values of the PTPET composites do not change greatly with the addition of OPS, PPSQ and PhVPOSS.

### 3.5. Swelling Properties

**Figure 12:**
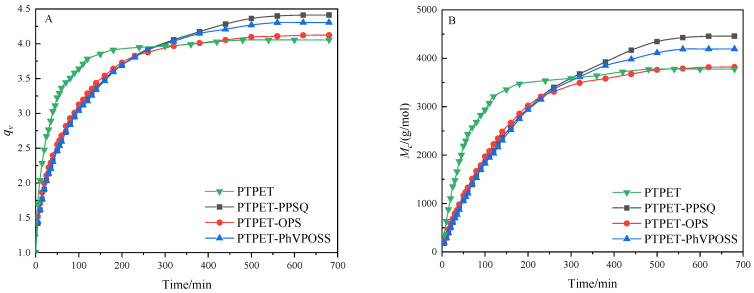
The volume swelling ratio (*q_v_*) (**A**) and apparent molecular weight (*Mc*) curves (**B**) of PTPET elastomer and composites.

PTPET elastomer and composites were swollen in toluene at 25 °C. Relative swelling parameters are calculated based on the equilibrium swelling method [27,28,29,30]. The volume swelling ratio (*q_v_*) and apparent molecular weight (*M_c_*) curves of PTPET composites are shown in Figure 12. The main parameters calculated based on the equilibrium swelling method are shown in Table 4. It could be seen from the swelling curves that all PTPET samples reached their swelling equilibrium at around 600 min. The swelling processes of the PTPET composites are roughly similar with some differences. The PTPET elastomer has the shortest time to reach swelling equilibrium, and the lowest volume swelling ratio and apparent molecular weight (*M_c_*). The apparent molecular weight (*M_c_* = 3820 g/mol) in the cross-linking network of PTPET-OPS composites is lower than that of PTPET-PPSQ and PTPET-PhVPOSS composites, whose apparent molecular weights are 4457 g/mol and 4192 g/mol, respectively. These indicate that the PTPET-OPS composite has a higher cross-linking network density and more cross-linking points in the system than the PTPET-PPSQ and PTPET-PhVPOSS.

### 3.6. Mechanical Properties

**Figure 13:**
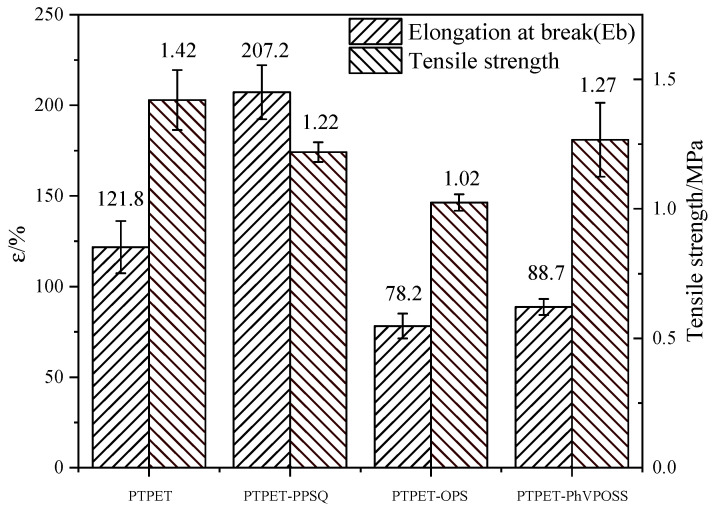
Tensile stress and strain of PTPET elastomer and composites.

The increase in the cross-linking network density in an elastomer can improve its mechanical strength [31], and the additives in the elastomer also have a great influence on the mechanical properties of the elastomer. The tensile test is believed to be an effective measurement to evaluate this influence. Detailed strain and stress data of the PTPET elastomer and composites are shown in Figure 13. It could be seen that the PTPET-PPSQ composite generally has the best mechanical performance. Its elongation at break is higher than that of the PTPET elastomer and the PTPET-OPS and PTPET-PhVPOSS elastomer composites, but its tensile strength is lower than that of PTPET elastomer and PTPET-PhVPOSS elastomer composite. This is due to the distinct toughening effects caused by different additives PPSQ, OPS and PhVPOSS.

### 3.7. SEM Observation

**Figure 14:**
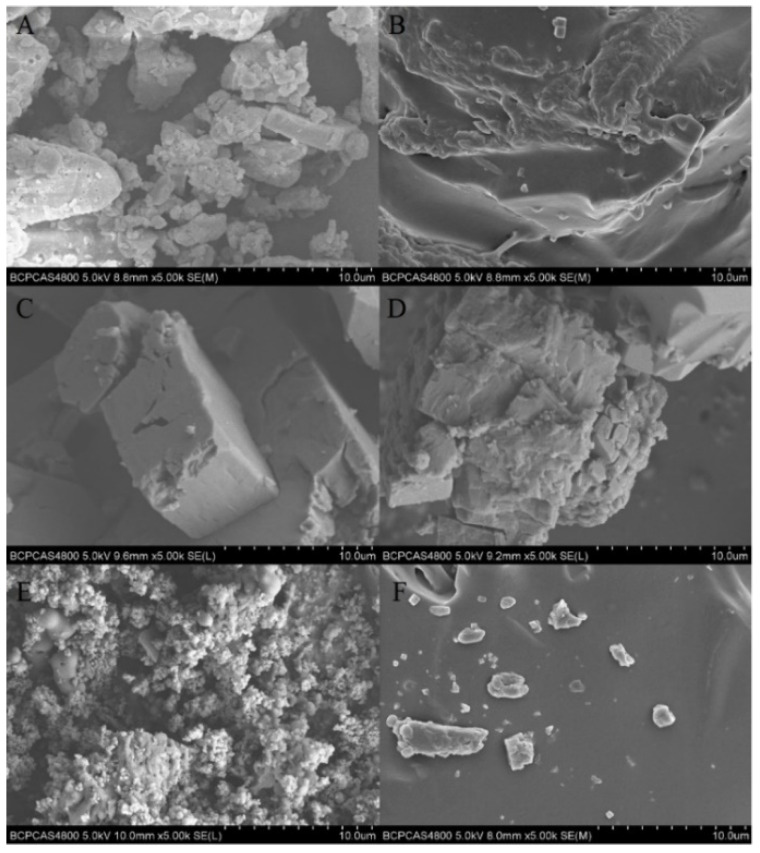
SEM photos of the additives and fracture morphology of PTPET composites: (**A**,**B**), PPSQ and PTPTE-PPSQ; (**C**,**D**) OPS and PTPET-OPS; (**E**,**F**) PhVPOSS and PTPET-PhVPOSS.

In order to analyze the morphology of the additives PPSQ, OPS and PhVPOSS in the PTPET composites and how these additives affect the properties of PTPET composites, the cross-sectional surfaces of PPSQ, OPS and PhVPOSS and the corresponding PTPET-PPSQ, PTPET-OPS and PTPET-PhVPOSS composites samples were observed by scanning electron microscopy (SEM) and are shown in Figure 14. It can be seen from Figure 14 that the particles of PPSQ and PhVPOSS are uniformly distributed in the PTPET matrix. The opposite is true for OPS. It can be seen from SEM that OPS agglomerates into irregular particles without good dispersion. This is consistent with the poor mechanical properties of PTPET-OPS and the better mechanical properties of PTPET-PPSQ and PTPET-PhVPOSS shown in Figure 13.

### 3.8. Flame Retardance

Cone calorimeter measurement is an effective method for obtaining numerous combustion data, e.g., ignition time (TTI), peak heat release rate (p-HRR), peak smoke generation rate (p-SPR) and mean CO and CO_2_ release (mean CO and CO_2_), to evaluate the flame retardancy of a material. Detailed data for the PTPET elastomer and PTPET-PPSQ, PTPET-OPS and PTPET-PhVPOSS composites are listed in Table 5. Table 5 shows that the ignition times of the PTPET composites are 12 s, 13 s and 14 s, respectively, which are longer than the 10 s of the PTPET elastomer. This is consistent with the thermal analysis results showing that the *T*_5%_ decomposition temperature and the maximum decomposition temperature *T_max_* of all three PTPET composites are higher than those of the PETPT elastomer.

**Figure 15:**
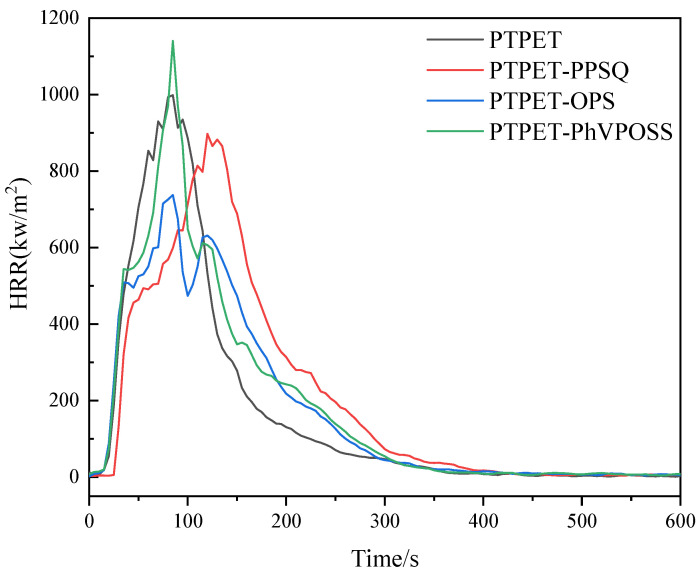
HRR curves of PTPET elastomer and composites.

The heat release (HRR) rate curves of the PTPET elastomer and composites are shown in Figure 15. It can be seen from Figure 15 that PTPET-PhVPOSS has a higher peak heat release rate (p-HRR) of 1140 kW/m^2^ soon after ignition, which is even greater than the 998 kW/m^2^ of the PTPET elastomer. The p-HRR of PTPET-PPSQ and PTPET-OPS composites exhibit only 897 kW/m^2^ and 737 kW/m^2^, respectively. For PTPET-OPS, two peaks appeared in the heat release (HRR) curve. It is believed that PTPET-OPS forms a carbon layer after the rapid combustion in the initial stage, which can reduce the heat release rate to a certain extent, but then the fragile and insufficiently compact carbon layer is subjected to continuous high temperature and oxidation during the combustion process and completely destroyed, so the heat release rate reaches a second peak. When comparing the cone test results of the three PTPET composites PTPET-PPSQ, PTPET-OPS and PTPET-PhVPOSS with the PTPET elastomer, it can be concluded that the addition of PPSQ and OPS can improve the flame retardancy of PTPET.

**Figure 16:**
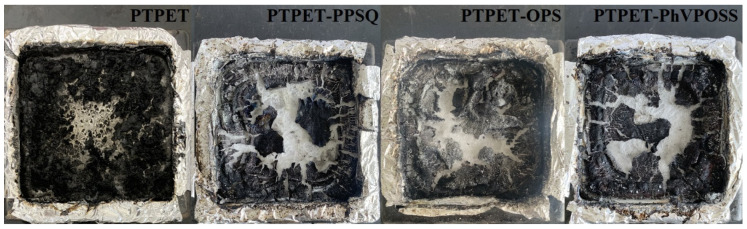
Carbon residues of PTPET elastomer and composites after cone test.

Figure 16 shows the residues of the PTPET elastomer and composites after the cone test. It seems that the pure PTPET elastomer has more char. The triazole structure in the PTPET network may be helpful to form some aromatic carbon during pyrolysis under high temperature. All the PTPET composites with the PPSQ, OPS and PhVPOSS form the cracked char layer after combustion. It is believed that all PTPET composites PTPET-PPSQ, PTPET-OPS and PTPET-PhVPOSS show certain flame retardancy, and PTPET-PPSQ and PTPET-OPS are better.

## 4. Conclusions

In this study, PTPET elastomer and composites PTPET-PPSQ, PTPET-OPS and PTPET-PhVPOSS with PPSQ, OPS and PhVPOSS as flame retardant additives were successfully prepared using the MiniLab twin-screw reactor for the first time. The reaction activation energy of the synthetic PTPET elastomer was then calculated based on the torque data from the MiniLab twin-screw reactor during the reaction process. It is concluded that MiniLab twin-screw reactor is an effective synthesis instrument. The click chemistry reaction is barely affected by the flame-retardant additives and could be catalyzed by the addition of Cu(I) catalyst C2610. The data obtained from the thermal analysis indicate that all PTPET composites have higher thermal stabilities and glass transition temperatures, which broaden the application environment of this engineering material. The mechanical tests show that PTPET-PPSQ has the highest tensile stress and strain. The results of the cone test show that the PTPET composites PTPET-PPSQ and PTPET-OPS prepared by adding flame retardants PPSQ and OPS have certain flame retardancy. Therefore, PTPET elastomers with different flame retardants are believed to be an option as additives in PTPET synthesis.

## Figures and Tables

**Table 1:** Formulation to prepare PTPET elastomer and composites.

Sample	Raw Materials Used in the Synthesis (g)					
	ATPET	GAP	C2610	PPSQ	OPS	PhVPOSS
PTPET	15	0.76	0.079	-	-	-
PTPET-PPSQ	15	0.76	0.079	1.6	-	-
PTPET-OPS	15	0.76	0.079	-	1.6	-
PTPET-PhVPOSS	15	0.76	0.079	-	-	1.6

**Table 2:** TG data of PTPET elastomer and composites.

Sample	*T*_5%_ (°C)	*T_max_* (°C)	Residue (%)
PTPET	292	394	7.73
PTPET-PPSQ	347	396	8.55
PTPET-OPS	351	396	19.33
PTPET-PhVPOSS	349	399	8.29

**Table 3:** DSC data of PTPET elastomer and composites.

Sample	*T_g_* (°C)
PTPET	−76
PTPET-PPSQ	−72
PTPET-OPS	−74
PTPET-PhVPOSS	−75

**Table 4:** Network parameters of PTPET elastomer and composites.

Samples	χ	*q_v_*	*V_p_*	*ρ* (g·cm^−3^)	*M_c_* (g·mol^−1^)	*N*_0_ (mmol·cm^−3^)
PTPET	0.34	4.05	0.247	1.113	3776	0.295
PTPET-PPSQ	0.34	4.412	0.227	1.083	4457	0.243
PTPET-OPS	0.34	4.124	0.243	1.083	3820	0.284
PTPET-PhVPOSS	0.34	4.305	0.232	1.077	4192	0.257

**Table 5:** Cone test data of PTPET elastomer and composites.

Sample	PTPET	PTPET-PPSQ	PTPET-OPS	PTPET-PhVPOSS
TTI (s)	10	14	13	12
p-HRR (kW/m^2^)	998	897	737	1140
THR (MJ/m^2^)	101	124.37	104.71	112
Mean CO	0.005	0.015	0.007	0.006
Mean CO_2_	0.555	1.93	0.836	0.78
p-SPR (m^2^/s)	0.013	0.039	0.0299	0.031
TSR (m^2^/m^2^)	152.7	519.76	438.5	421
Residues (%)	5.9	3.93	3.96	4.1

## Data Availability

The data that support the findings of this study are available from the corresponding author upon reasonable request.
